# Books and Magazines

**Published:** 1905-01

**Authors:** 


### Books and Magazines.

THE DOCTOR'S RECREATION SERIES. Charles Wells Moulton, general editor. Published by the Saalsfield Publishing Company, Akron, O. Facts and fancies of interest to the doctor and his patient. Arranged by Porter Davis, M. D. Price per volume, cloth, $2.50; leather, $4. 352 pages.

We have received another volume of this delightful series. It is a whole story and not selections and extracts like the first two volumes. I say "story;" it is not a novel, nor is it a story. It appears to be an autobiography of one "Dr. Brush," who practiced medicine in a village in Maine in the year 1800. And yet it is not a connected or complete biography. A better title would have been "Recollections of a Doctor of 1800." It is by Dr. W. S. Kelley, Cleveland, O. Call it what you will, it is delightful reading. It is full of thrilling events, brightened by occasional bits of sentiment and philosophy. It holds the reader's interest from start to finish, notwithstanding the introduction of matter irrelevant to the story proper, and of unimportant incidents. There is a love story and a terrible tragedy running through it, and, while this is so often interrupted, as stated, the interest is keen to find out more about the villain in the plot, and we would be tempted to skip but for the charm of the style of narration, so simple and so elegant. It reminds one of that beautiful little classic, "Vicar of Wakefield." One almost believes he is reading the narrative of old Dr. Primrose. Dr. Kelley is to be congratulated on his production. I surely enjoyed it. Every doctor will enjoy it. It was in the days before chloroform, the days of antimony and the lancet, when "tractoration" *a la* Perkins, was a fad, but not with Brush.

---

SIMOND'S PHYSIOLOGICAL CHEMISTRY.—A Text-Book of Physiological Chemistry.—For students and practitioners of medicine. By Charles E. Simon, M. D., late resident physician, Johns Hopkins Hospital; author of Simon's Clinical Diagnosis, etc. New (2d) edition. Revised and enlarged. Octavo, 500 pages. Cloth, $3.25, net. Lea Brothers & Co., Publishers, Philadelphia and New York.

Dr. Simon has here treated Physiological Chemistry in a manner adapting his work to the wants of the medical student, and of the physician who has previously been unable to devote to the subject the attention which it merits. It deals with foods, their origin, classes and decomposition products, their digestion, resorption and excretion, the chemistry of the tissues and organs of the body, the substances resulting from their activity and their relation to physiological function. The early call for a new edition has enabled the author to include the results of the very active research in this field to date. The chapters on the Albumins, Nitrogenous Katabolism and Gastric and Tryptic Digestion have been rewritten. To render the work still more useful both to students and teachers, laboratory exercises have been added. The methods have been described in such detail that the student should find no difficulty in performing the experiments.

---

SAUNDER'S QUESTION COMPENDS—ESSENTIALS OF MEDICAL CHEMISTRY—Containing also questions on Medical Physics, Chemical Philosophy, Medical Processes, Toxicology, etc. By Lawrence Wolff, M. D., formerly Demonstrator of Chemistry at the Jefferson Medical College, Philadelphia. Sixth edition, thoroughly revised by A. Ferree Witmer, Ph. G., formerly Assistant Demonstrator in Physiology at the University of Pennsylvania. 12mo volume of 225 pages, fully illustrated. Philadelphia, New York, London: W. B. Saunders & Co., 1904. Cloth, $1, net.

We need but mention the fact that this little book has reached its sixth edition to prove beyond question its practical usefulness. The recent important discoveries in physics and inorganic chemistry have rendered it necessary, in Dr. Witmer's revision, to make extensive additions almost to every part of the work. The subject of organic chemistry, especially organotherapy and the substituted ammonias, has also been carefully revised and much new matter added. We find the book unusually excellent.

---

EXAMINATION OF THE URINE. By G. A. de Santos Saxe, M. D., Pathologist to the Columbus Hospital, New York City. 12mo volume of 391 pages, fully illustrated, including eight colored plates. Philadelphia, New York, London: W. B. Saunders & Co., 1904. Flexible leather, $1.50, net.

Dr. Saxe has presented a work on examination of the urine unusually complete, absolutely up-to-date, concise, yet explicit in all its parts; and it will be found to meet fully the requirements of the student and practitioner without burdening him with unnecessary analytic procedures. Special attention has been paid to the interpretation of findings as applied to clinical diagnosis, and the student is told what each chemical element and each microscopic structure means when found in the urine. The character of the urine in various diseases is also described in detail. Descriptions of technic have been made very explicit, and the author has inserted some new methods of working developed in his own experience. Cystos copy and other means of functional diagnosis have been given their proper places. The text is fully illustrated, including eight colored plates of the various urinary crystals. The work will be useful because it is practical.

---

A TEXT-BOOK OF CLINICAL DIAGNOSIS—By Laboratory Methods—For the Use of Students, Practitioners, and Laboratory Workers. By Napoleon Boston, A. M., M. D., Associate in Medicine and Director of the Clinical Laboratories of the Medico-Chirurgical College, Philadelphia; formerly Bacteriologist at the Philadelphia Hospital and at the Ayer Clinical Laboratory of the Pennsylvania Hospital. Octavo volume of 547 pages, with 320 illustrations, many of them in colors. Philadelphia, New York, London: W. B. Saunders & Co., 1904. Cloth, $4, net; sheep or half Morocco, $5, net.

Dr. Boston here presents a practical manual of those clinical laboratory methods which furnish a guide to correct diagnosis, giving only such methods, however, that can be carried out by the busy practitioner in his office as well as by the student in the laboratory. He has given special attention to outlining in progressive steps the various procedures in clinical technic, such steps being illustrated whenever possible. All the more recent methods for the examination and staining of blood are described and illustrated by original drawings, and the subject of Serum-Diagnosis is very carefully considered. The newer methods for the estimation of sugar, Bence-Jones' albumin, uric acid, and purin have received thoughtful consideration. The subjects of Animal Parasites, Diseases of the Skin, Transudates, and Secretions of the Eye and Ear have received an unusual amount of space. Attention has also been paid to Inoscopy and Cyto-diagnosis. Indeed the book contains useful material throughout, and being the latest work on Clinical Diagnosis, includes the most recent advances along that line.

---

HAND-BOOK OF THE ANATOMY AND DISEASES OF THE EYE AND EAR—For students and practitioners. By D. B. St. John Roosa, M. D., LL. D., Professor of Diseases of the Eye and Ear in the New York Post-Graduate Medical School; formerly President of the New York Academy of Medicine, etc., and A. Edward Davis, A. M., M. D., Professor of Diseases of the Eye in the New York Post-Graduate Medical School; Fellow of the New York Academy of Medicine. Three hundred pages, square 12 mo. Price, extra cloth, $1, net. F. A. Davis Company, Publishers, 1904-16 Cherry Street, Philadelphia, Pa.

---

ESSENTIALS OF ANATOMY; INCLUDING THE ANATOMY OF THE VISCERA. By Charles B. Nancrede, M. D., Professor of Surgery and Clinical Surgery in the University of Michigan, Ann Arbor. Seventh edition, thoroughly revised. 12mo volume of 419 pages, fully illustrated. Philadelphia, New York, London: W. B. Saunders & Company. 1904. Cloth, $1.00 net.

This work, now in its seventh edition, has met with a most cordial reception. In this revision the entire book has been thoroughly gone over and the section on the Nervous system completely rewritten. The illustrations throughout the text are excellent, showing the anatomy of various parts with unusual clearness. Students, and indeed young practitioners, will find the work of great service.

---

We have before us "Practical Dietetics with Reference to Diet in Disease." This book is published by Alida Frances Pattee. 12mo; cloth; 300 pages. Price, $1.00 net, by mail; C. O. D., $1.25. A. F. Pattee, Publisher, 52 West Thirty-ninth Street, New York. It is a text-book for the physician, student and nurse, giving in detail the method of preparing and administering liquid, semi-liquid and solid food to patients and convalescents. It appears clear to us that if the mothers of our land would use this book there would be less cause for a physician and nurse in our homes.

